# A Systematic Review of Screening Tests for Chronic Kidney Disease: An Accuracy Analysis

**DOI:** 10.31661/gmj.v9i0.1573

**Published:** 2020-06-22

**Authors:** Fatemeh Keshvari-Shad, Sakineh Hajebrahimi, Maria Pilar Laguna Pes, Alireza Mahboub-Ahari, Mohammad Nouri, Farshad Seyednejad, Mahmood Yousefi

**Affiliations:** ^1^Department of Health Economics, School of Management and Medical Informatics, Tabriz University of Medical Sceinecs, Tabriz, Iran; ^2^Research Center for Evidence Based Medicine, Faculty of Medicine, Urology Department, Tabriz University of Medical Sciences, Tabriz, Iran; ^3^Department of Urology Istanbul Medipol University Istanbul, Turkey; ^4^Department of Health Economics, Iranian Evidence-Based Medicine Center of Excellence, School of Management and Medical Informatics, Tabriz University of Medical Sciences, Tabriz, Iran; ^5^Department of Biochemistry and Clinical Laboratories, Tabriz University of Medical Sciences, Tabriz, Iran; ^6^Department of Radiation Oncology, Madani Hospital, Tabriz Medical University, Tabriz, Iran; ^7^Department of Health Economics, Iranian Center of Excellence in Health Management, School of Management and Medical Informatics, Tabriz University of Medical Sciences, Tabriz, Iran

**Keywords:** Chronic Kidney Disease, Screening, Sensitivity, Specificity, Systematic Review

## Abstract

This systematic review was conducted to assess the diagnostic accuracy of chronic kidney disease screening tests in the general population. MEDLINE, EMBASE, Web of Science, Scopus, The Cochrane Library and ProQuest databases were searched for English-language publications up to November 2016. Two reviewers independently screened studies and extracted study data in standardized tables. Methodological quality was assessed using the QUADAS-2 tool. Sensitivity and specificity of all available screening methods were identified through included studies. Ten out of 1349 screened records included for final analysis. Sensitivities of the dipstick test with a cutoff value of trace were ranged from 37.1% to 69.4% and specificities from 93.7% to 97.3% for the detection of ACR>30 mg/g. The diagnostic sensitivities of the UAC>10 mg/dL testing was shown to vary from 40% to 87%, and specificities ranged from 75% to 96%. While the sensitivities of ACR were fluctuating between 74% and 90%, likewise the specificities were between 77% and 88%. Sensitivities for C-G, Grubb and Larsson equations were 98.9%, 86.2%, and 70.1% respectively. In the meantime the study showed specificities of 84.8%, 84.2% and 90.5% respectively for these equations. Individual studies were highly heterogeneous in terms of target populations, type of screening tests, thresholds used to detect CKD and variations in design. Results pointed to the superiority of UAC and dipstick over the other tests in terms of all parameters involved. The diversity of methods and thresholds for detection of CKD, necessitate considering the cost parameter along with the effectiveness of tests to scale-up an efficient strategy.

## Introduction

Chronic Kidney Disease (CKD) is one of the leading causes of mortality and morbidity throughout the world. The prevalence of CKD (stages 1-5) has been estimated around 13.4% worldwide [1]. CKD annually imposes a significant economic burden on health systems and societies [2,3]. In 2002, the National Kidney Foundation-Kidney Disease Outcomes Quality Initiative (NKF-KDOQI) published the first guideline and defined the CKD as kidney damage or kidney dysfunction (estimated glomerular filtration rate [eGFR]<60 mL/min/1.73 m2) that lasts for at least three months [4]. The CKD often, until its late stages, is silent and asymptomatic. Evidence shows that the early detection of CKD based on the presence of proteinuria or reduced eGFR can prevent or delay the progression of the disease to advanced stages [5]. The considerable burden of the CKD, along with the availability and effectiveness of diagnostic tests, and treatments for early detected CKD patients, makes the condition as an appropriate candidate for the screening [6]. By realizing the fact that both the general and high-risk population will theoretically benefit from the undergoing of CKD screening programs [7], different strategies of CKD screening for detecting patients with CKD have been developed. The most common tests for the diagnosis of CKD include GFR, which is estimated through the serum creatinine concentration (eGFR) and albuminuria, which is measured by the urinary albumin to creatinine ratio (ACR) [8-11]. The diversity of existing diagnostic strategies necessitates the understanding of the strengths and limitations of each diagnostic approach to go through efficient decision making [12]. Since screening targets people with apparently healthy conditions, the test should be applied to a large proportion of the population [13-15]. Thus it can be argued that the initiation of a screening program requires a significant amount of society’s resources should be allocated to the program [16-18]. In other words, any decision about CKD screening in favor of society requires examining all the available options [19]. Accordingly, the decision-makers need high-quality data to support decisions about a diagnostic test in the screening program. Understanding the accuracy of each screening intervention in terms of sensitivity and specificity is essential for reaching a rigorous conclusion on the decisions made [20], such that the uncertainty in each of these parameters will affect the final outcome. Addressing the abovementioned issues, the aim of this systematic review is to find and extract information on sensitivity and specificity of CKD screening tests in the general population in a way that makes the application of results in screening programs feasible.

## Search Strategy

###  Study Selection

We followed the Preferred Reporting Items for a Systematic Review and Meta-analysis of Diagnostic Test Accuracy Studies (PRISMA-DTA) guidelines for conducting and reporting systematic reviews [21]. We performed a comprehensive search of MEDLINE (PubMed), EMBASE, Web of Science, Scopus, the Cochrane Library, and ProQuest databases up to November 2016 and updated later to the end of 2017. The search strategy included three major key terms: screening, CKD, and screening tests for CKD. Furthermore, a combination of words such as “screening,” “albuminuria,” “proteinuria,” “glomerular filtration rate,” “creatinine,” “Chronic kidney disease,” “Chronic renal disease,” “Chronic renal insufficiencies,” “Chronic renal failure,” “Chronic Kidney Failure” were searched using each individual databases. We also used the Medical Subject Headings (MeSH) terms in the search strategy, and the search was limited to the English language. Using the EndNote X7.4, a pool of retrieved literature was constructed. By removing the duplicates, the title and abstract of the remained studies screened by two independent reviewers (F.K and M.Y). In the cases where relevant studies might have been missed due to the improper search strategy, a list of the article references as well as the related systematic reviews were also checked in full-text by the reviewers. Any disagreement was resolved through consensus. It is worth mentioning that different study designs were incorporated into this review including those with one or more index tests and with any reference method (gold standard) that investigated the CKD screening in the general population. Eligible studies had to report sensitivity and specificity or the data that could be used to calculate those values, involve an asymptomatic population, included adult populations, and be published as full-length articles. Studies that reported outcomes from diabetic or hypertension groups were excluded.

###  Data Extraction and Quality Assessment

Two reviewers (F.K and M.Y) independently extracted the relevant data using a created data extraction form. The following data was captured from studies; characteristics of the studies such as publication date and location, study sample, the type of study, age-range and mean age, index test, reference test, threshold level, and outcome measures such as sensitivity, specificity and likelihood ratios (LRs). The quality of included studies was assessed using the Quality Assessment of Diagnostic Accuracy Studies-2 (QUADAS-2) tool by two independent reviewers [22]. It consists of four key domains, including patient selection, index test, reference standard, and flow of patients and timing of the index test and reference standard. The risk of bias and applicability concerns were assessed using a number of signaling questions for each study. Disagreements about the risk of bias and applicability concerns in each domain were resolved with the arbitration of the third and fourth investigator (S.H and A.M).

###  Analysis

Sensitivity, specificity, and LRs were descriptively analyzed for the included studies. Sensitivity is defined as the percentage of individuals with the disease that correctly identified, and specificity as the percentage of the individual without disease that correctly identified [23]. For studies in which positive and negative LR (PLR and NLR) had not been reported, these values were calculated as follows: PLR=sensitivity/ (1-specificity); and NLR=(1-sensitivity)/specificity. The LR specify how many times more likely, it is that to receive a particular test result in people with target condition than without [24]. Given that the study aimed at finding all available strategies of CKD screening then there was a great heterogeneity in the target populations, types of tests, thresholds used and variations in the design of included studies this made doing the meta-analysis of effect size inappropriate.

## Results

###  Study Selection and Characteristics

**Figure 1:**
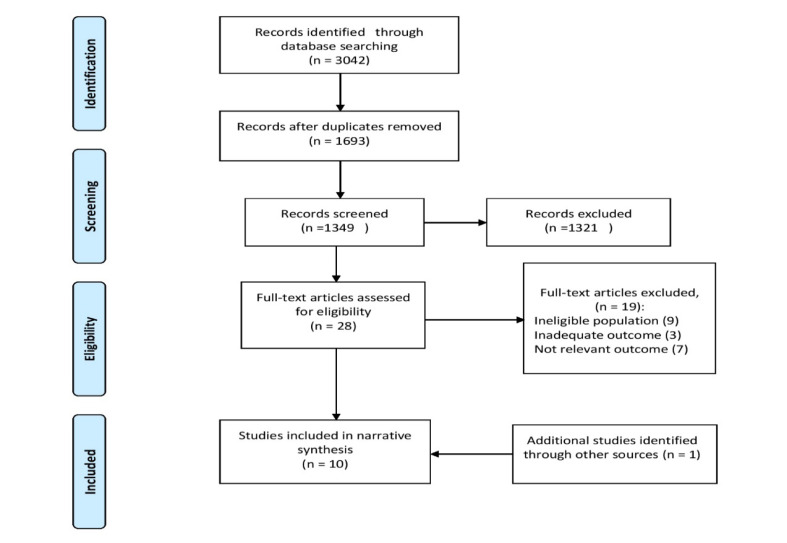
Flowchart of the article selection process.

A total of 3042 citations were initially identified. After removing duplicates, 1349 results were screened based on title and abstract, out of which 28 full texts were identified to be examined (Figure-1). Finally, nine studies met the review criteria, and 19 studies were excluded due to not meeting the inclusion criteria. One further study was identified by the updated search in MEDLINE (PubMed) and included in this review [25]. In total, ten articles were included in this review. Eight out of ten selected studies had a cross-sectional design [25-32]. One was a cohort study [33] and one study was a cross-sectional cohort [34]. These studies had been published from 2005 to 2017 with worldwide distribution, including china, Australia, Netherlands, Japan, Pakistan, Taiwan, Italy, Iceland, and South Korea. General characteristics of the selected studies are summarized in Table-1. Briefly, these studies have included population samples ranging from 557 to 43,516 participants. The mean age of the subjects was between 43 to 59.7 years. Except for two studies [29,30], gender distribution was described in all studies [25-28,32-34]. Nine studies had been conducted on general the population, and one study included diabetic patients as well [30]. It was demonstrated that age is an indispensable part of all studies and had been considered as inclusion criteria.

###  Index and Reference Tests

In order to detect CKD, different studies had utilized various screening tests. The eGFR was evaluated in one study [32]. Three studies used the dipstick test for detection of albuminuria [25,26,34]. Strip test was used as an index test for measuring the ACR in one study [30]. Three of the ten included studies evaluated the urine albumin concentration (UAC) [27,28,33], two of which also made a comparison of the UAC and ACR [27,28]. One article provided separate assessments of semi-quantitative urine protein-to-creatinine (P/C) ratios, quantitative protein concentrations, and dipstick protein [29]. One study assessed routine urinalysis [31]. The ACR was used as the reference standard in three studies [25,26,34]. GFR was used in one study [31]. Three studies considered the 24-hour urine collection UAE ≥30 mg as the reference test [27,28,33]; and the rest of the studies used quantitative P/C ratio and laboratory method in urine as the reference standard [29,30]. Except for one study [32], the reference standard and the procedures were adequately described in most of the included articles.

###  Study Quality

**Figure 2:**
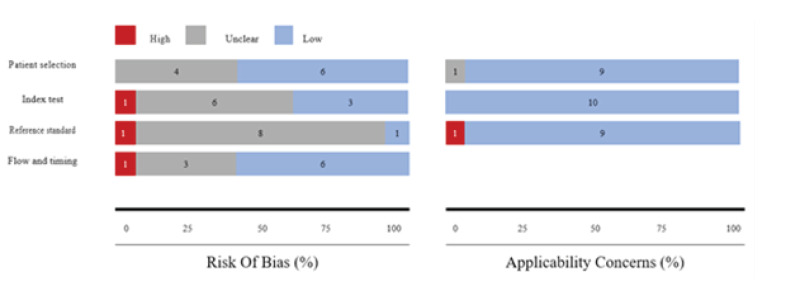
Bar charts for QUADAS-2 analysis. Risk of bias and applicability concerns graph review investigators’ judgments about each domain presented as percentages across included studies.

In general, the data showed a satisfactory level of quality for the selected studies. Nine studies exhibited a low or unclear risk of bias as well as applicability concerns. Moreover, most of the studies demonstrated a clear description of the subjects, index and the reference tests, and diagnostic criteria (Figure-2). Due to the ambiguous methods of patient selection, four studies were identified to have presented an unclear risk of bias in patient selection [25,26,29,31]. The risk of bias primarily arose from insufficient blinding between the index and reference tests [25,26,28,29,31]. Also, high risk of bias was observed in one study [32] in which no standard test was specified. Three studies also failed to demonstrate a clear interval between the index and reference tests [26,27,30].

###  Diagnostic Accuracy

A high degree of heterogeneity was found between studies in terms of reported sensitivity and specificity of included index tests. The sensitivity, specificity, and LRs for each study have been summarized in Table-2. The accuracy of dipstick testing was evaluated across the general population in three studies [25,26,34]. For the detection of ACR>30 mg/g, the sensitivities of the dipstick with a cut-off point of trace were ranged from 37.1-69.4% and specificities from 93.7-97.3%. We have also obtained 23.3% to 98.9% sensitivities and 92.6% to 98.9% specificities for the dipstick test result of >1 and identified ACR of >300 mg/g (massive proteinuria). The study by Graziani *et al*. [30], was the only study that evaluated the test accuracy of a strip test for measuring ACR, where they used a cut-off of 3.4 mg/mmol to define microalbuminuria in the general population and to compare it with those found in a diabetic population. The test results of this study demonstrated a sensitivity and specificity of 92 % and 95 %, respectively. Furthermore, in the diabetic group, the sensitivity and specificity of the test was 92 % and 95 %, respectively. The UAC was examined in three selected studies [27,28,33]. The diagnostic sensitivities of the UAC>10 mg/dL testing were shown to range from 40% to 87%, whereas the specificities ranged from 75% to 96%. Two studies demonstrated that the sensitivities of ACR varied between 74% and 90%, and the specificities ranged between 77% and 88% [27,28]. One study examined the performance of routine urinalysis for the diagnosis of eGFR<60 ml/min/1.73 m2 [31]. The sensitivity and specificity of urinalysis were 11% and 92/8% respectively. Wetmore *et al*. compared the performance of “C-G,” “Grubb” and “Larsson” equations with the “Modification of Diet in Renal Disease (MDRD)” equation to eGFR, with a cut-off point of 60 ml/min/1.73 m2. The sensitivity for C-G, Grubb and Larsson equations was 98.9%, 86.2%, and 70.1%, respectively. The study also showed the specificities of 84.8%, 84.2%, and 90.5% for these equations, respectively. The C-G equation had better performance in terms of sensitivity and specificity. Semi-quantitative P/C ratio, dipstick protein, and quantitative protein tests were compared in one study for detecting proteinuria [29]. For Semi-Quantitative P/C ratio sensitivities were 70-75.6%, and specificity was 95.9% to both of them. Sensitivity and specificity for dipstick protein were 45.0% and 98.3%, respectively. Also, the study reported the accuracy of the quantitative protein test, for which a sensitivity of 50.1% and a specificity of 98.2% was reported.

## Discussion

In the current study, we systematically reviewed the literature to evaluate the accuracy of different tests for screening CKD among the general population without risk factors for CKD. Although little evidence exists on the recommendation of routine screening [7,14,35], guidelines propose the detecting of urine protein (micr- or macro albuminuria) as well as measuring the serum creatinine to estimate GFR for the screening of CKD [8,36,37]. Despite the availability of a wide range of screening tests, selecting a single method, and defining the specific criteria for further implications remain to be major consideration [7,38,39]. The present study is one of the pioneering systematic reviews, which compares the diagnostic accuracy of various tests for CKD screening in the general population. To obtain more insights into the accuracy of the tests for CKD, ten studies were included in our review. Overall, a broad range of sensitivity and specificity was reported for the various tests. The variations in index and reference tests, threshold, participants, and study designs among the studies do not allow for performing a meta-analysis of the data. Our findings highlighted that the UAC test, with high sensitivity and specificity, can indeed compete with the ACR to accurately detect microalbuminuria across the general population in 24-hour timed urine collections as the gold standard. Sensitivities above 74% and specificities above 81% were reported for the ACR and the UAC. However, no significant difference was observed in the ability of the UAC and the ACR to detect microalbuminuria [27,28]. Generally, the ACR has been accepted to offer a slightly better diagnostic accuracy than measuring solely the concentration of urine albumin to detect albuminuria in many populations. This can be due to the composition variability in the standardization of the methods used for quantifying total protein in urine samples. However, in terms of the cost, this method is more expensive in comparison with methods used for total urine protein measurement and decisions on the recommendation of this strategy needs other criteria to be taken into account [8,40]. In this systematic review, when the estimation of the accuracy of urine dipstick by comparing its characteristics to spot ACR as the gold standard is considered, three studies showed poor sensitivity and high specificity [25,26,34]. Due to its unclear clinical significance, the result of trace protein reading on urinalysis on the general population is mostly disregarded by the clinicians [41,42]. However, proteinuria is considered as an independent risk factor to develop end-stage renal disease [43]. Despite this, two studies have supported the concomitant occurrence of trace proteinuria and microalbuminuria in a large proportion of individuals, especially men, the elderly, diabetic patients, and patients with hypertension. As well, these studies revealed that using the trace as a cut-off value led to recovery both in terms of sensitivity and specificity [26,34]. A high sensitivity and specificity was shown by Graziani *et al*. in which the strip test was used to measure the ACR in the general population [30]. The current review has several strength points that include presenting the methods used for the identification and recruitment of the available literature, as well as using the most up to date guidelines for diagnostic reviews. We performed a comprehensive systematic review of six electronic data bases and continuously adapted the review during the writing process. We exclusively considered studies that performed on the general population. Selected studies incorporate a wide spectrum of demographic characteristics from Asia, Europe, and Australia supporting the generalizability of their results. In this review, the details of the index test, reference test, and population characteristics were deemed to have been adequately reported. The overall quality of original studies was also assessed, pointing to minimal risk of bias and applicability concerns. There are several limitations in our study. First, this review only includes studies published in English that may cause language bias. Second, the attempt to have the advantage of accessing to all available options led to an increase in heterogeneity between different screening methods, which in turn prevented conducting a meta-analysis. The weak points mostly rooted in the methodological constraints of the included studies, especially the blinding of operators when conducting and interpreting the index and reference tests. Differences in gender, race, and prevalence of CKD between studies could also contribute to some of the variability in the study results. In this review, the female participants of the included studies were mostly older adults fluctuating on a wide range from 36-63.8%. The selected studies had also compared various tests available in local laboratory methods. In most of the cases, large biases occur in the existing laboratory methods. For instance, although testing the total protein using 24-hours urine collections is the gold standard for comparing proteinuria assays, it has several limitations such as being time consuming, cumbersome, inconvenience for patients. Furthermore, errors such as incomplete collection may lead to inaccuracies [44,45]. To the best of our knowledge, no systematic review has been previously conducted to assess the diagnostic performance of various screening tests for CKD risk in the general population. A recent review on diabetic patients reported that either UAC or ACR can yield a similar sensitivity and specificity to detect microalbuminuria. The findings of the aforementioned study concluded that the UAC and ACR can offer rational rule out results to detecting significant proteinuria in diabetic patients [46]. There are also still issues ahead of using CKD screening in settings where limited resources are available [7,47]. Nevertheless, depending on the availability of resources and the level of risks (e.g., diabetic patients and the general population) different results are expected in terms of cost effectiveness of CKD screening [48,49]. In addition, there is still a lack of strong guidelines specifically addressing the CKD screening in general population and resource-limited settings [50]. In a systematic review published by Fink *et al*. studying the RCT of CKD screening, no direct evidence was found to confirm the advantages or disadvantages of CKD screening or monitoring of patients with stages 1-3 of CKD progression [51]. While indirect evidence proposed that targeting CKD screening or monitoring may be possible but the potential benefit of these interventions was not ensured. A major standard for an accurate screening test is the acceptable sensitivity, specificity, and high predictive values [52-54]. The better the performance of the test, the higher is the chance of detecting disease. This reduces the burden of false positive results, which can lead to additional detriment and costs [7,55]. The screening tests usually burden various levels of false positive results, and thus may dramatically influence the results taken from subjects where the prevalence of disease is very low [56]. The dipstick screening method has numerous well-known potential benefits including feasibility and potential to be used as a test for CKD screening in resource-limited settings [57]. However, urine dipstick testing fails to meet the whole criteria of an ideal screening test [52] and it may burden many false positive results when conduction on the general population (between 53.1% and 72.8% of positive tests for detection of ACR>30 mg/g), leading to over-diagnosis of many CKD high-risk group when the diagnostic tests are not repeated [34]. This also poses an economic concern, since it increases the unnecessary therapeutic interventions or further diagnostic investigations where the resources are almost inadequate. In conclusion, we conducted a systematic review to assess the diagnostic accuracy of CKD screening tests in the general population. According to our results, the UAC and ACR yielded high sensitivity and specificity in the general population and the diagnostic performance of the UAC is similar to ACR for accurate detection of microalbuminuria in general population, but less expensive. Therefore, the UAC may become the screening tool of choice for the general population. Regarding sensitivity and specificity of urine dipsticks in this review, dipstick proteinuria has been suggested as a CKD screening test in resource-limited settings.

## Conclusion

 Further studies are needed to evaluate the accuracy of CKD screening tests in the general population. The choice of an effective screening tool for detection of CKD requires a comprehensive evaluation of all possible strategies in terms of accuracy measures, threshold levels and the quality of conducted studies. Given the diversity of the screening methods as well as the availability of various thresholds for detection of CKD, requires considering the cost parameter along with the effectiveness of tests to scale-up an efficient strategy. UAC and dipstick revealed superiority over the others when it comes to considering all parameters together. But for choosing between these two tests in population-scale, it needs the affordability issue to be taken into account and cost of implementing each strategy be compared in terms of the cost-effectiveness.

## Acknowledgment

 This study was part of a master degree thesis supported by the Tabriz University of Medical Sceinecs (IR.TBZMED.REC.1396.135).

## Conflict of Interest

 The authors declare that they have no Conflict of interest.

**Table 1:** Characteristics of Studies Included in the Systematic Review

**Study, Year **	**Country**	**Population characteristics**	**Study setting**	**Study design**	**Sample size**	**Age ( Mean±SD )**	**Female (%)**	**Definition of CKD**
White *et al*., 2011[34]	Australia	Australian adults 25 years and older and high-risk subgroups	AusDiab, a representative survey of Australian adults 25 years and older (conducted in 1999/2000)	Cross-sectional cohort	10944	51.6 ± 14.4	54.7%	ACR≥30 mg/g or ACR≥300 mg/g
Park *et al*., 2017[25]	South Korea	general population >20	The Korean National Health and Nutrition Examination Survey (KNHANES)	Cross-sectional survey	20759	46.6	48%	ACR≥30 mg/g or ACR≥300 mg/g
Konta *et al*., 2007[26]	Japan	general population >40	Community-based health check-up in Takahata, Japan	Cross-sectional	2321	64	55.5%	ACR≥30 mg/g or ACR≥300 mg/g
VanderVelde *et al*., 2010[33]	Netherlands	General population (28–75 years)	Prevention of Renal and Vascular End-stage Disease (PREVEND) Study	Cohort study	3398	49	55%	UAE ≥30 mg
Gansevoort *et al*., 2005[27]	the Netherlands	General population (28–75 years)	Prevention of Renal and Vascular End-stage Disease (PREVEND) study	Cross-sectional	2527	48.8	52.9%	UAE ≥30 mg
Jafar et a,l. 2007[28]	Pakistan	General population (>40 years)	Cohort study of Population-Based Strategies for Effective Control of High Blood Pressure in Indo-Asian, Pakistan	Cross-sectional	577	51.8	54.4%	UAE ≥30 mg
Chang *et al*., 2016[29]	Taiwan	Taiwanese aged at least 40 years and participating in regular physical examinations	Regular physical examinations, the National Health Insurance Administration, Ministry of Health and Welfare, Taiwan	Cross-sectional	2932	_	_	Proteinuria (150 mg protein/g creatinine)
Graziani *et al*., 2009[30]	Italy	general population, diabetic patients	The ‘INCIPE’ study (Initiative on Nephropathy of relevance to public health, which is Chronic, possibly in its Initial stages, and carries a Potential risk of major clinical End-points)	Cross-sectional	GP :201 DP:259	_	_	(ACR [cut-off <3.4mg/mmol])
Xue *et al*., 2016[31]	China	Healthy adults who underwent physical examination between September 2008 an September 2013	Physical examinations (PE) during a health check-up at Zhongshan Hospital, between September 2008 in china	Cross-sectional	43516	43	36.7%	eGFR (<60ml/min/1.3 m 2)
Wetmore *et al*., 2010[32]	Iceland	general population	A study on bone health in community-dwelling Icelandic adults between January 2001 and January 2003	Cross-sectional	1628	59.7 ± 14.8	63.8%	eGFR (<60ml/min/1.3 m 2)

**GP**: General population; **DP**: Diabetic patient

**Table 2:** Characteristics of the Screening Tests and Summary of Diagnostic Accuracy in Studies

**Study, Year**		**Index test**	**Reference standard**	**Sensitivity** **(%)**	**Specificity** **(%)**	positive	**Likelihood ratio** negative
White *et al*., 2011[34]	Dipstick	≥1+proteinuria	ACR≥30 mg/g	57.8	95.4	12.57	0.40
			ACR≥300 mg/g	98.9	92.6	13.36	0.019
		≥trace proteinuria	ACR≥30 mg/g	69.4	86.8	5.26	0.33
			ACR≥300 mg/g	100	83.7	6.14	0
Park *et al*., 2017[25]	Dipstick	≥1+proteinuria	ACR≥300 mg/g	75.4	99.5	157.93	0.25
		≥trace proteinuria	ACR≥30 mg/g	43.6	93.6	6.85	0.6
Konta *et al*, 2007[26]	Dipstick	≥1+proteinuria	ACR≥300 mg/g	23.3	98.9	21.18	0.77
		≥trace proteinuria	ACR≥30 mg/g	37	97.3	13.7	0.65
VanderVelde *et al*., 2010[33]	UAC	>20 mg/ L	24-hour urine collection UAE ≥30 mg >10 mg/ L High CV risk High CV risk+ age >55	40	96	10.08	0.62
				58	81	3.12	0.51
				28	90	2.95	0.79
				65	71	2.25	0.49
Gansevoort *et al*., 2005[27]	UAC	24-hour urine collection UAE ≥30 mg	UAC: AUC 0.92, DV 11.2 mg/L	85	85	5.67	0.17
	ACR		AUC 0.93, DV 9.9 mg/g	87.6	87.5	7.00	0.14
Jafar *et al*., 2007[28]	UAC	Female	24-hour urine collection UAE ≥30 mg	UAC: AUC 0.86, DV 0.5 mg/dL	87	74.9	3.47	0.17
		Male			73.9	93.6	11.54	0.27
	ACR	Female		UAC: AUC 0.86, DV 1.7 mg/dL	89.2	81	4.7	0.13
		Male			90	76.9	3.9	0.13
Chang *et al*., 2016[29]	Semi-quantitative P/C ratio (excluding diluted samples)		Quantitative P/C ratio (150 mg)	75.6	95.9	18.43	0.25
		Semi-quantitative P/C ratio (including diluted samples)	70	95.9	17.07	0.31
		Dipstick protein	45	98.3	26.47	0.56
		Quantitative protein	50.1	98.2	27.84	0.50
Graziani *et al*., 2009[30]	Strip test	Laboratory method (ACR [cut-off<3.4mg/mmol])	General population	90	91	10	0.10
			Diabetic group	91	92	11.33	0.09
Xue *et al*., 2016[31]	Routine urinalysis	eGFR (<60 ml/min/1.73 m ^2^)	11	92.8	1.53	0.96
Wetmore *et al*., 2010[32]	eGFR<60 ml/min/1.73 m^2^	Equation C-G	98.9	84.8	7.19	0.012
		Grubb equation	86.2	84.3	5.5	0.16
		Larsson equation	70.1	90.5	7.38	0.33

**ACR**: Albumin- creatinine ratio; **UAC**: Urinary albumin concentration; **AUC**: Area under the curve; **DV**: Discriminator value; **CV**: Cardiovascular; **UAE**: Urinary albumin excretion; **eGFR**: Estimated glomerular filtration rate; **C-G equation**: Cockroft–Gault equation; **P/C**: Protein- to- creatinine
